# Investigation of the Physico-Chemical Properties that Enable Co-Formulation of Basal Insulin Degludec with Fast-Acting Insulin Aspart

**DOI:** 10.1007/s11095-014-1614-x

**Published:** 2015-01-08

**Authors:** Svend Havelund, Ulla Ribel, František Hubálek, Thomas Hoeg-Jensen, Per-Olof Wahlund, Ib Jonassen

**Affiliations:** 1 Diabetes Formulation, Biophysics and Structure, Novo Nordisk A/S, Novo Nordisk Park D6.1126, DK-2760 Maalov, Denmark; 2 Clamp Competency Center, Novo Nordisk A/S, Novo Nordisk Park, DK-2760 Maalov, Denmark; 3 Yeast and Protein Technology, Novo Nordisk A/S, Novo Nordisk Park, DK-2760 Maalov, Denmark; 4 Diabetes Peptide and Protein Chemistry, Novo Nordisk A/S, Novo Nordisk Park, DK-2760 Maalov, Denmark

**Keywords:** co-formulation, insulin analog, insulin aspart, insulin degludec

## Abstract

**Purpose:**

To study the self-association states of insulin degludec and insulin aspart alone and combined in pharmaceutical formulation and under conditions simulating the subcutaneous depot.

**Methods:**

Formulations were made of 0.6 mM degludec at 3 and 5 Zn/6 insulin monomers, and 0.6 mM aspart (2 Zn/6 insulin monomers). Self-association was assessed using size-exclusion chromatography (SEC) monitored by UV and orthogonal reverse-phase chromatography.

**Results:**

Simulating pharmaceutical formulation, degludec eluted as dihexamers, whereas aspart eluted as hexamers and monomers. Combining degludec at low zinc with aspart increased dihexamer content, indicating hybrid hexamer formation. At high zinc concentration, however, there was no evidence of this. Simulating the subcutaneous depot by removing preservative, degludec eluted as multihexamers and aspart as monomers. Aspart was incorporated into the multihexamer structures when combined with degludec at low zinc, but there was no such interaction with high-zinc degludec. SEC using progressively diluted concentrations of phenol and m-cresol showed that dissociation of aspart into monomers occurs before the formation of degludec multihexamers.

**Conclusion:**

Insulins degludec and aspart can be combined without forming hybrid hexamers, but this combinability is dependent on zinc and preservative concentration, and requires that degludec is fully dihexameric before addition of aspart.

## Introduction

Insulin naturally associates into a hexameric structure in the presence of zinc ions, thought to be an adaptation for efficient storage within pancreatic beta-cell vesicles [1]. Upon secretion into the circulation, dilution causes the insulin hexamers to quickly dissociate into dimers and the biologically active monomers. In normal physiology, insulin secretion is closely regulated, typically presenting as a slow and relatively steady ‘basal’ rate, periodically supplemented by greatly increased peaks of secretion in response to nutrient ingestion [2].

While self-association of insulin is physiologically beneficial, it poses problems for subcutaneous injection therapy for diabetes. Hexamers are relatively large structures that do not diffuse across capillary membranes as readily as monomers do, and to provide a therapeutic insulin dose in a tolerable injection volume requires insulin to be formulated at concentrations at which it is hexameric [3]. This means that a subcutaneous depot is formed in which dilution occurs slowly, hence the dissociation of hexamers and absorption of insulin into the circulation is retarded, rising to and falling from a peak over a period of a few hours. The resultant pharmacokinetic (PK) profile resembles neither natural basal insulin secretion nor the prandial response [3]. To better recreate natural patterns of plasma insulin kinetics, analogs of insulin have been developed. Thus, rapid-acting insulin analogs contain minor modifications to the amino acid sequence in regions involved in self-association that are designed to weaken insulin–insulin binding and accelerate absorption, producing a kinetic profile similar to the natural prandial response [3]. An example is insulin aspart (B28 aspartic acid human insulin) in which proline is substituted by aspartic acid at the B28 position.

To recreate the basal level of circulating insulin, several principles have been applied, all of which serve to increase the molecular mass/self-association state of the insulin in the depot. In the case of neutral protamine Hagedorn (NPH) insulin, the peptide protamine is added to the formulation causing the insulin to form a crystalline precipitate that dissolves after injection [4]. NPH has been widely used, but its kinetic profile is an imperfect approximation of basal insulin secretion, with absorption still tending to rise and fall from a peak concentration (C_max_) value [5–7]. Furthermore, its absorption rate is rather unpredictable, and this problem is compounded by the need to re-suspend the precipitate prior to injection, which patients may not do effectively [8, 9]. Two soluble insulin analogs improve on this profile. Insulin glargine (marketed as Lantus® by Sanofi) is presented as a solute in a slightly acidic formulation, but it precipitates after injection as pH rises [10]. Insulin detemir (marketed as Levemir® by Novo Nordisk A/S) features the attachment of a myristic acid side chain, which enables reversible albumin binding. This, together with dihexameric self-association, prolongs the absorption profile [11].

Each of these basal insulin analogs can be used to supplement or recreate physiological basal insulin availability, and rapid-acting analogs can be given additionally at mealtimes to supplement or recreate the prandial insulin response. Such basal–bolus insulin regimens are, however, challenging for patients as they require multiple daily injections with frequent blood glucose sampling to ensure optimal dose adjustment. This is of vital importance in type 1 and advanced type 2 diabetes where the regimen constitutes full insulin-replacement therapy but, in earlier stages of type 2 diabetes, simpler, more convenient regimens may suffice and aid adherence.

A popular option in type 2 diabetes is to use a ‘premixed’ insulin formulation whereby a single injection can be given with main meals to supplement both prandial and basal insulin output. Ideally, this would be a mixture of two insulin analogs designed specifically for these roles, but there are problems: rapid-acting analogs require neutral formulations to remain stable, while insulin glargine requires an acidic formulation to remain soluble; hence, this mixture is considered incompatible. Indeed, a clamp study has shown that the pharmacodynamic (PD) profile of insulin lispro is flattened when combined with glargine [12]. Insulin detemir can be co-formulated in a neutral environment with other insulins, but there are self-association equilibriums for these components that could enable monomers to recombine in hybrid hexamers. Again, a clamp study has shown that the peaked PD profile of insulin aspart is attenuated and prolonged when combined with detemir [13], consistent with hybrid hexamer formation. Until now, premixed insulin products have therefore reverted to the protamination technology used for NPH insulin. Thus, human insulin or a rapid-acting analog is formulated with sufficient protamine to crystallize a percentage (e.g., 70%) of the insulin content. This then acts as the basal component, with the remaining insulin solute absorbing rapidly to provide a prandial dose [3]. Unsurprisingly, however, such products bring with them the kinetic limitations associated with NPH insulin [8, 14]. Adequate resuspension is required to ensure dose accuracy, and the absorption kinetics of the basal component is faster and with more of a peak effect than would be desired.

Insulin degludec (N^ε^-hexadecandioyl-γ-Glu-Lys^B29^ desB30 human insulin) is the latest basal insulin analog to be marketed. It differs from human insulin by deletion of the B30 threonine and addition by acylation at B29 with a glutamate spacer and 16-carbon fatty diacid side chain. The protraction mechanism involves post-injection formation of long multihexamer chains from which monomers are slowly released [15, 16], producing a flat, glucose-lowering action at steady state, with lower intra-patient day-to-day variability than insulin glargine [17]. In pharmaceutical formulation, in the presence of preservatives including phenol, insulin degludec assembles as dihexamers held together by side chain–zinc contacts, a structure considered to be highly stable [15, 16]. It is therefore feasible that insulin degludec could be combined in solution with a fast-acting insulin such as insulin aspart without interactions occurring in the formulation. After injection, phenol diffuses quickly from an insulin injection depot [11], and this process causes insulin degludec to undergo the conformational change required to enable multihexamer formation [15]. If the post-injection formation of insulin degludec multihexamers and the disassembly of insulin aspart hexamers were then to be discrete processes, the individual PK/PD profile of the two insulin analogs should then be preserved, giving a clearer separation of the basal and prandial kinetics than hitherto possible with traditional premix products. We therefore investigated the association states of these insulin analogs (in isolation and co-formulated) in conditions designed to simulate the pharmaceutical formulation and subcutaneous depot to assess the extent of any interactions between insulin degludec and insulin aspart.

## Materials and Methods

Insulin degludec was prepared as described earlier [18]. Formulations were made by dissolution of insulin degludec followed by addition of 1.6% glycerol, 16 mM phenol, 16 mM m-cresol, 10 mM sodium chloride, and zinc acetate so as to create ratios of 3 or 5 Zn ions per 6 insulin monomers (Zn/6ins). The pH was adjusted to 7.4, and water added to final volume with an insulin degludec concentration of 0.6 mM.

Insulin aspart and human insulin were obtained at Novo Nordisk, Denmark. Formulations were made by suspension of 0.6 mM insulin analog and dissolution by addition of diluted hydrochloric acid followed by zinc acetate to 2 Zn/6ins, 1.6% glycerol, 16 mM phenol, 16 mM m-cresol, 10 mM sodium chloride, and pH adjusted to 7.4 by diluted sodium hydroxide. Formulations of 0.6 mM insulin degludec at 3 and 5 Zn/6ins were combined with 0.6 mM insulin aspart and human insulin respectively in proportions of 70:30 and stored for 4 weeks at 25°C. For comparison, Levemir® (insulin detemir) was combined with NovoRapid® (insulin aspart) in the same proportions and stored for 4 weeks at 25°C.

## Size-Exclusion Chromatography

Size-exclusion chromatography (SEC) was performed to characterize the association states for the individual basal and rapid-acting insulins, and for the combinations. Three SEC methods were used as *in vitro* models to simulate conditions in the pharmaceutical formulation (at high concentration of phenol and room temperature), the condition in the subcutaneous depot after injection (without phenol and at body temperature) and, finally, to evaluate a serial decrease in concentration of phenol and m-cresol, as expected to occur following subcutaneous injection. Furthermore, fractions of high and low molar mass were collected from the SEC eluents, and reverse-phase chromatography was used to measure the concentrations of the individual insulin within them.

SEC method 1 was designed to simulate conditions in the pharmaceutical formulation (with the same phenol concentration), and was used to measure the percentage of oligomers of dihexamer, dihexamers, hexamers, and monomers with eluent of 16 mM phenol, 140 mM sodium chloride, 10 mM tris (hydroxymethyl) aminomethane (tris) pH 7.3, and 0.01% sodium azide at 23°C. The size-exclusion column used was ACQUITY UPLC® BEH200 (150*4.6 mm, *d* = 1.7 μm) from Waters Corporation, Milford, MA, USA. Ultraviolet detection wavelengths were at 286, 276 and 290 nm for methods 1–3, respectively. Injection volume was 20 μL and flow was 0.15 mL/min.

SEC method 2 was designed to simulate conditions in the subcutaneous depot (in which phenol quickly dissipates), and was used to measure the percentage of multihexamer *versus* the hexamer–monomer fraction with a phenol-free eluent of 140 mM sodium chloride, 10 mM tris pH 7.3, 0.01% sodium azide, and 5% 2-propanol at 37°C. The concentrations of the individual insulin analogs in the multihexamer fraction and the hexamer–monomer fraction were determined by reverse-phase chromatography. A SymmetryShield RP18 (3.9*20 mm, d = 3.5 μm) column from Waters Corporation (Milford, MA, USA) was eluted with A: 10%(vol) acetonitrile 0.2 M sodium sulfate, 40 mM o-phosphoric acid adjusted to pH 3.6 with sodium hydroxide and B: 70%(vol) acetonitrile at a gradient of 20–54% at 1.4–5 min at 30°C, 1 mL/min, and detection at 276 nm. To avoid adsorption, 70 ppm polysorbate 20 was added to the fraction vials.

For comparison, insulin detemir was combined with insulin aspart in the same proportions (volume 70:30) and stored for 4 weeks at 25°C. SEC method 2 was modified for the combination of insulin detemir and insulin aspart by dividing fraction collection between hexamer and dimer (since insulin detemir achieves its protracted absorption largely through reversible albumin binding rather than multihexamer formation).

SEC method 1 was not employed for an insulin detemir and insulin aspart combination. This is because in the phenolic SEC eluent (similar to the pharmaceutical formulation), insulin detemir elutes as a hexamer [11, 15], as does insulin aspart, so the two insulin analogs (and any hybrid associations) would therefore be indistinguishable using this method.

SEC method 3 was designed to simulate serial conditions at decreasing concentrations of the preservatives phenol and m-cresol, as will occur immediately after subcutaneous injection. These tests were made by mixing A: 16 mM phenol and m-cresol, 20 mM sodium chloride, 3 mM sodium dihydrogenphosphate pH 7.3, and 0.01% sodium azide, with B: 140 mM sodium chloride, 3 mM sodium dihydrogenphosphate pH 7.3, and 0.01% sodium azide at 37°C. Transformation of insulin degludec dihexamers to multihexamers, and of insulin aspart hexamers to monomers, was compared with human insulin.

## Dynamic Light Scattering

Dynamic light scattering (DLS) also provides information about the molecular size of solutes and was used to validate the SEC results by testing the formulations prepared for SEC method 1. These insulin formulations were expected to contain insulin in various self-association states, hence the acquired hydrodynamic radius for each formulation will be an average (R_h average_). Since the insulin concentration was kept constant to 0.6 mM in all formulations, the average hydrodynamic radii are comparable. DLS data were acquired at 25°C by adding 30 μL of each sample in triplicates to a 384 well plate Corning® 3540 (Corning, NY, USA). Each well was measured 20 times with 10 s acquisition time, resulting in 600 measurements for each sample composition. A DynaPro™ Plate Reader and Dynamics™ software version 7.1.8.93 (Wyatt Technology Corp., Santa Barbara, CA, USA) was used to collect and analyse the data.

## Results

### Pharmaceutical Formulation Simulations

**Fig. 1:**
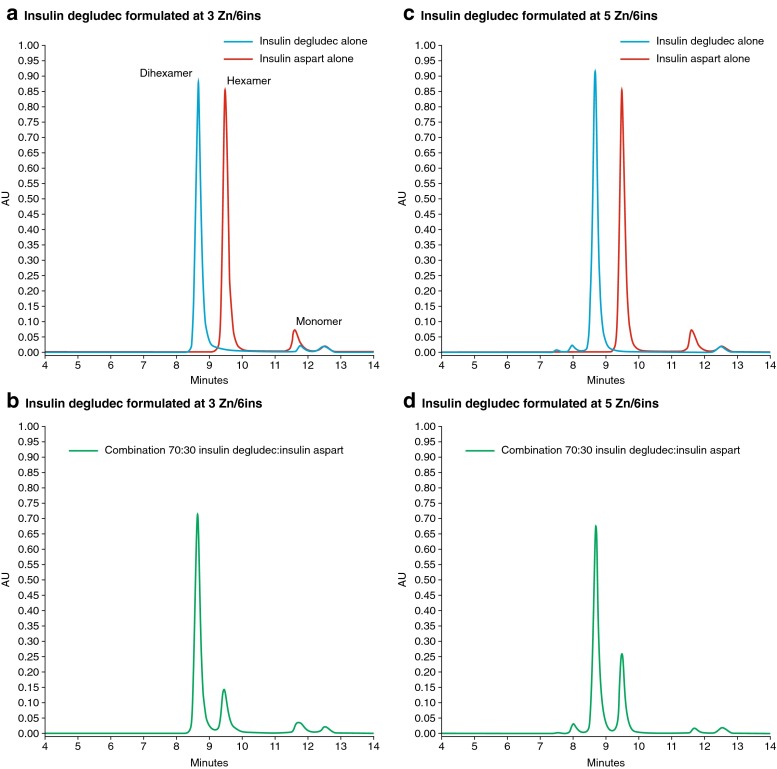
SEC simulating pharmaceutical formulation (method 1). Elution curves comparing insulin analogues eluted alone or in combination, with insulin degludec formulated at two different zinc (Zn) concentrations (n zinc ions per 6 insulin monomers). When mixed together in a 70:30 ratio, the proportion of insulin eluting as dihexamers is increased beyond 70% at the lower (**b**), but not the higher (**d**), zinc concentration. *AU*, absorbance unit; *SEC*, size-exclusion chromatography.

Data for the self-association states of the various insulin preparations eluted using SEC method 1 are presented in Table I and Fig. 1. When formulated with a zinc concentration of 3 Zn/6ins, insulin degludec eluted primarily in a dihexameric state (91%) with a fraction of hexamers and monomers. When zinc was increased to 5 Zn/6ins, insulin degludec eluted mostly as dihexamers or larger structures. Insulin aspart at 2 Zn/6ins eluted as hexamers (88%) and monomers, and human insulin (at the same zinc concentration) eluted primarily as hexamers (98%), with the remainder as monomers. For the combinations of insulin degludec and insulin aspart (prepared in a 70:30 ratio), the proportions of dihexamer *versus* hexamer–monomer were 73:27 and 71:29 at the two insulin degludec formulation zinc concentrations of 3 and 5 Zn/6ins, respectively. A similar set of experiments substituting insulin aspart with human insulin showed proportions of dihexamer *versus* hexamer–monomer of 87:13 and 74:26, respectively.

**Table I:** SEC and DLS Simulating Pharmaceutical Formulation (Method 1). Self-Association States for Individual and Combined Insulin Preparations Formulated with Pharmaceutical Preservatives and Different Zinc (Zn) Concentrations Determined by SEC, with Hydrodynamic Radius (R_h_) and Polydispersity Index (PDI) Determined by DLS. Combined Formulations Included 70% Insulin Degludec and 30% Insulin Aspart or Human Insulin, Stored for 4 weeks at 25°C. DLS Data on Unperturbed Formulations are Included

Insulin/zinc formulation		% eluting as dihexamer or larger structure in SEC	% eluting as hexamer or smaller structure in SEC	R_h average_ in DLS	PDI in DLS
Insulin degludec	3 Zn/6ins alone	91	9	3.04 ± 0.06	0.15 ± 0.06
Insulin degludec	5 Zn/6ins alone	100	0	3.15 ± 0.01	0.22 ± 0.01
Insulin aspart	2 Zn/6ins alone	0	100	2.47 ± 0.00	0.24 ± 0.00
Human insulin	2 Zn/6ins alone	0	100	2.52 ± 0.00	0.24 ± 0.00
Insulin degludec Insulin aspart	3 Zn/6ins + 2 Zn/6ins	73	27	3.05 ± 0.02	0.23 ± 0.00
Insulin degludec Insulin aspart	5 Zn/6ins + 2 Zn/6ins	71	29	3.04 ± 0.01	0.14 ± 0.02
Insulin degludec Human insulin	3 Zn/6ins + 2 Zn/6ins	87	13	2.95 ± 0.04	0.13 ± 0.03
Insulin degludec Human insulin	5 Zn/6ins + 2 Zn/6ins	74	26	3.30 ± 0.04	0.22 ± 0.01

*DLS*, dynamic light scattering; *SEC*, size-exclusion chromatography

### Subcutaneous Depot Simulations

**Fig. 2:**
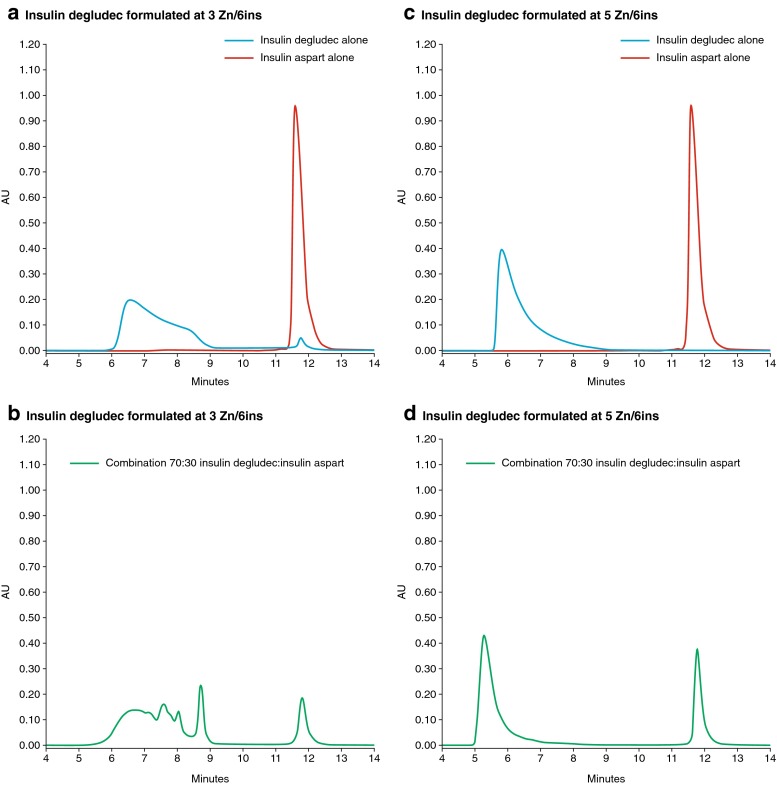
SEC simulating depot environment (method 2). Elution curves comparing insulin analogs eluted alone or in combination with insulin degludec formulated at two different zinc (Zn) concentrations (n zinc ions per 6 insulin monomers). When mixed together in a 70:30 ratio, the proportion of insulin aspart eluting as monomers is reduced at the lower zinc concentration (**b**) due to incorporation into multihexamers. At the higher zinc concentration (**d**), insulin aspart elutes almost entirely in the monomeric fraction, and insulin degludec elutes in the multihexameric fraction, which shows slight leftward shift, indicating the formation of larger multihexamers. *AU*, absorbance unit; *SEC*, size-exclusion chromatography.

Data for the self-association states of the insulin preparations eluted using SEC method 2 are presented in Table II and Fig. 2. Using SEC method 2 (without phenol), insulin degludec eluted as multihexamers, whereas insulin aspart was fully monomeric and human insulin eluted below hexamer size. A high molar mass and a low molar mass fraction were collected from the eluent (dividing between dihexamer and hexamer), and the concentrations and proportions of the constituent insulin were determined by reverse-phase chromatography (Table II). When eluted alone, insulin degludec primarily appeared in the multihexamer fraction (91% at 3 Zn/6ins and 99% at 5 Zn/6ins), whereas insulin aspart and human insulin were only found in the monomer–hexamer fraction when eluted alone.

**Table II:** SEC Simulating Subcutaneous Environment (Method 2). Percentages of Insulin Type Recovered from Eluent Fractions Distinguished by Mass for Individual and Combined Insulin Preparations Formulated with Pharmaceutical Preservatives and at Different Zinc (Zn) Concentrations. Combined Formulations Included 70% Insulin Degludec and 30% Insulin Aspart or Human Insulin, Stored for 4 weeks at 25°C. Eluents were Phenol-Free and Warmed to 37°C

Insulin/zinc formulation		% of total insulin degludec eluting in multihexamer fraction	% of total insulin aspart/human insulin eluting in hexamer or smaller fraction
Insulin degludec	3 Zn/6ins alone	91	–
Insulin degludec	5 Zn/6ins alone	99	–
Insulin aspart	2 Zn/6ins alone	–	100
Human insulin	2 Zn/6ins alone	–	100
Insulin degludec Insulin aspart	3 Zn/6ins + 2 Zn/6ins	89	35
Insulin degludec Insulin aspart	5 Zn/6ins + 2 Zn/6ins	99	99
Insulin degludec Human insulin	3 Zn/6ins + 2 Zn/6ins	92	8
Insulin degludec Human insulin	5 Zn/6ins + 2 Zn/6ins	100	88

The combination of insulin degludec and insulin aspart at low zinc concentration showed 89% of insulin degludec to be in the multihexamer fraction and 35% of aspart in the monomer–hexamer fraction. Substituting insulin aspart with human insulin, the distributions were 92 and 8% respectively. At the higher zinc concentration, however, 99% of insulin aspart appeared in the monomer–hexamer fraction, with a similar proportion of insulin degludec eluting as multihexamers. For human insulin, 88% appeared in the monomer–hexamer fraction when combined with insulin degludec in the 5 Zn/6ins concentration (Table II).

In the insulin detemir and insulin aspart simulations, insulin detemir, when eluted alone, appeared mainly in a dihexamer–hexamer fraction (86%), whereas insulin aspart alone eluted at a size smaller than hexameric (99%). When insulin detemir and insulin aspart were combined, 82% of insulin detemir was found in the dihexamer–hexamer fraction, whereas the content of insulin aspart eluting below hexamer size was reduced to 64%.

### Simulations of Serial Decrease in Preservative Concentration

**Fig. 3:**
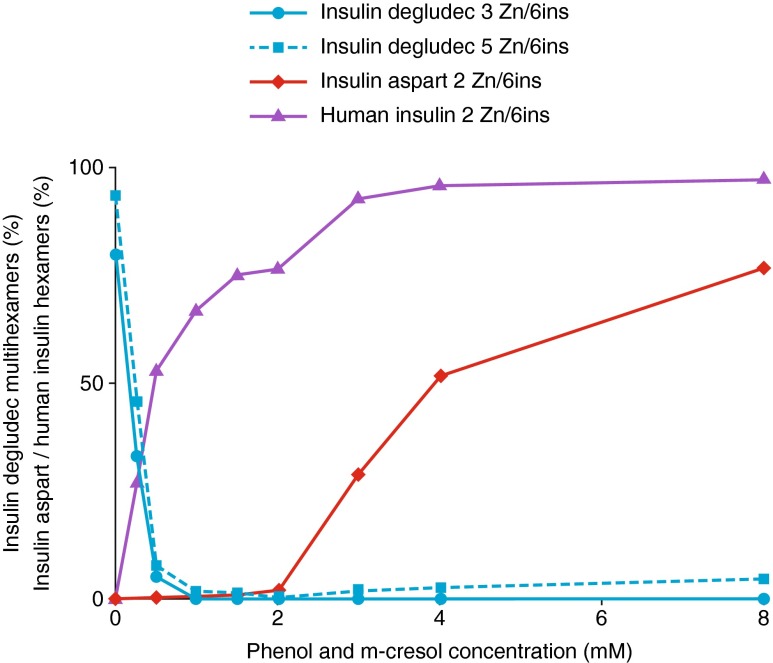
SEC simulating depot environment (method 3), with varied pharmaceutical preservative concentrations (phenol and m-cresol). The percent of insulin aspart eluting as hexamers declines quickly as preservative concentration decreases, while insulin degludec becomes multihexameric only at very low phenol and m-cresol concentration. *SEC*, size-exclusion chromatography; *Zn*, zinc.

In the SEC method 3 tests, insulin degludec eluted mainly as dihexamers until the preservative concentration dropped to a threshold of 0.5 mM phenol and m-cresol, at which point self-association into multihexamers began to occur (Fig. 3). Insulin aspart eluted mainly as hexamers when the preservatives were at a concentration of 8 mM, but it became fully monomeric at the lower preservative concentration of 2 mM phenol and m-cresol (Fig. 3). Finally, human insulin remained hexameric at lower preservative concentrations than insulin aspart, but eluted below hexameric size when phenol and m-cresol were absent.

### Validation of SEC Data by DLS

DLS data are summarized in Table I. Formulations of insulin degludec with 5 Zn/6ins had a slightly larger average hydrodynamic radii (R_h average_) compared with 3 Zn/6ins. The R_h average_ for insulin aspart and human insulin (2 Zn/6ins) were smaller than those of insulin degludec and similar to each other. In the two co-formulations of insulin degludec and insulin aspart, the R_h average_ values were almost identical. The R_h average_ of the co-formulation with 5 Zn/6 insulin degludec and human insulin was, on the other hand, significantly larger than the formulation containing 3 Zn/6 insulin degludec and human insulin.

## Discussion

The SEC studies simulating the pharmaceutical formulation show that insulin degludec is primarily self-associated as dihexamers, with a small monomer fraction at low zinc concentrations (3 Zn/6ins) or a minor tetrahexamer content at high zinc concentration (5 Zn/6ins). These observations are therefore consistent with earlier SEC studies [15]. In a pharmaceutical formulation, the short-acting analog, insulin aspart, and human insulin were shown to be mainly self-associated as hexamers, but with some monomeric content at low zinc concentration (2 Zn/6ins). Combining insulin degludec at a low zinc concentration with insulin aspart (70:30 ratio) showed a dihexamer content that was higher than the proportion of insulin degludec. This suggests that degludec–aspart hybrid hexamers (and thereby hybrid dihexamers) were forming, presumably through the association of free monomers of insulin degludec and insulin aspart. When human insulin was combined with insulin degludec, the dihexamer content was higher still. A combination of insulin degludec at the high zinc concentration with insulin aspart, however, showed a dihexamer content corresponding almost exactly to the proportion of insulin degludec, although an increased amount of dihexamer was still seen with human insulin. Generally, an increased zinc concentration induces increased insulin self-association, but in this experiment the increase in zinc is actually associated with less dihexamer formation. The likely explanation is that when degludec is formulated at higher zinc concentration, monomers are no longer present, hence degludec–aspart hybrid hexamers can no longer form when insulin aspart is added. These results can be interpreted as showing that insulin degludec and insulin aspart can be co-formulated at high zinc concentrations with little, if any, interaction occurring between the two, provided insulin aspart is added to fully dihexameric insulin degludec. With human insulin, however, a co-formulation does still result in the occurrence of some hybrid self-associations, which might be explained by the relatively greater tendency for human insulin to self-associate compared with insulin aspart.

We consider that the DLS data support these interpretations. Formulations of insulin aspart and human insulin had R_h average_ values that were consistent with the hydrodynamic size of an insulin hexamer at low ionic strength [19, 20], whereas the insulin degludec formulations had R_h average_ values in the expected range for an insulin solution comprising mainly dihexamers [19]. The polydispersity index (PDI) indicates that species of different sizes are present in solution. The SEC data show that the co-formulations contain structures ranging from monomer to > dihexamer size, so the R_h average_ values for the co-formulations are difficult to interpret in detail. For the two insulin aspart plus insulin degludec co-formulations, however, the similar radii are consistent with the fact that ≥ dihexamer:≤ hexamer ratios obtained by SEC were almost identical. The co-formulation with 3 Zn/6 insulin degludec and human insulin had a smaller R_h average_ than the formulation containing 5 Zn/6 insulin degludec and human insulin. Hence, the size distribution appears to be shifted towards larger species. This might seem to contradict the SEC results (Table I) where the fraction eluting as ≤ hexamer is increased, while the fraction eluting as ≥ dihexamer is decreased as we move from 3 to 5 Zn/6 insulin degludec; if a larger fraction of ≤ hexamer and a smaller fraction with species ≥ dihexamer are formed, then intuitively the expectation is of a decrease in overall average size. However, this expectation is based on the assumption that the internal size distribution of the two fractions remains identical. In fact, as explained above (and illustrated in Fig. 1) an increased zinc concentration will result in the increased association of monomers and dimers into hexamers, and furthermore, dihexamers start to associate as tetrahexamers (more pronounced for the degludec and human insulin co-formulation). Therefore, the R_h average_ of the overall value is increased.

The subcutis simulations were designed to test whether interactions can still occur between the different insulin components following injection. Eluting each individual insulin alone showed the self-association state it would naturally adopt with the dissipation of preservatives. Consistent with previous research [15, 16], insulin degludec eluted primarily as high-mass multihexameric complexes, while insulin aspart eluted in a monomer–hexamer fraction. When insulin degludec and insulin aspart were combined in a low zinc concentration, only 35% of the insulin aspart eluted in the monomer–hexamer fraction, implying that much of it was being incorporated into the multihexamer complexes and/or that hybrid dihexamers were again forming. This was also true for human insulin. When a higher zinc concentration was used, however, the interaction between insulin degludec and insulin aspart again disappeared, while the interaction between insulin degludec and human insulin was diminished. Interestingly, in the insulin degludec and insulin aspart combination with high zinc, the elution curve for insulin degludec was shifted slightly to the left, implying the formation of larger multihexamer complexes (Fig. 2d). A possible explanation for this observation is that the additional zinc ions liberated from dissociated insulin aspart hexamers enable insulin degludec to form into larger multihexamer units, since zinc binding is a characteristic of hexameric but not of monomeric insulin.

Insulin detemir was also studied alone and in combination with insulin aspart and these studies showed clear differences between the self-association states when the two insulin analogs were eluted alone compared with when they were combined. In the latter situation, the percentage of insulin aspart eluting in the monomer–hexamer fraction decreased from 99 to 64%, implying that about a third of the insulin aspart was associating into dihexameric structures with insulin detemir.

The serial (method 3) SEC study, with diminishing concentrations of preservatives, was designed to examine the relative time frames over which insulin degludec would adopt a multihexamer self-association state, and insulin aspart and human insulin would become monomeric. The relevance of this is that, as part of the process of forming multihexamer chains, insulin degludec hexamers undergo a conformational change (as phenol dissipates) whereby both poles of the hexamer open to allow linkage between a zinc ion in the core of one hexamer and a fatty diacid side chain of another hexamer [15, 16]. At this point in the linking process, it would be theoretically possible for hexamers of another insulin to be incorporated into the multihexamer chains as a result of interactions between their zinc ions and insulin degludec side chains. However, our studies showed that insulin aspart becomes completely monomeric at a higher preservative concentration than that at which insulin degludec begins to form multihexamers. This was not true, however, for human insulin.

**Fig. 4:**
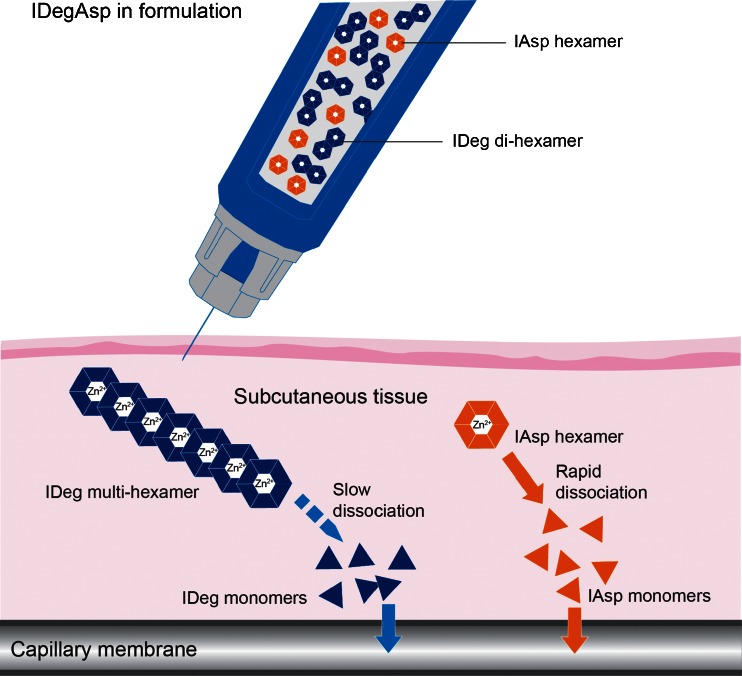
After injection, insulin degludec and insulin aspart undergo different changes. Insulin aspart hexamers rapidly dissociate into monomers that are readily absorbed into the circulation over a short time. Insulin degludec dihexamers associate to form soluble multihexamers from which monomers are slowly released to be absorbed into the circulation over a prolonged time. *IAsp*, insulin aspart; *IDeg*, insulin degludec.

Therefore, the subcutaneous simulations collectively show that, in the high zinc concentration formulation, the self-association state kinetics of insulin degludec and insulin aspart will take place independently of each other after injection (Fig. 4). In contrast, there is a degree of interaction between insulin degludec and human insulin.

## Conclusion

Our studies collectively demonstrate how hybrid insulin associations can form when different insulin molecules are co-formulated, especially at lower zinc concentrations, and also how hybrid associations can form in conditions that simulate the subcutaneous depot. This problem has hitherto prevented the co-formulation of two different insulin analogs that have distinct and desired PK/PD profiles (e.g., insulin detemir and insulin aspart) because these profiles would have been compromised by such interactions. Therefore, ‘premix’ insulin analog products have all been based on partial protamination of a rapid-acting insulin. However, our studies have also shown that at the high zinc concentration used in the pharmaceutical formulation, insulin degludec and insulin aspart do not interact in conditions that simulate either the formulation or the subcutaneous depot. Furthermore, the serial SEC study suggests that the two processes of insulin aspart hexamer dissociation and insulin degludec multihexamer formation will occur sequentially and separately, thereby preventing another potential interaction. These studies therefore predict that the discrete PK/PD profiles of insulin degludec and insulin aspart would be preserved in a high zinc co-formulation, and this expectation has indeed been borne out in a clamp study [14], and in clinical studies of the combination product, as evidenced by clinical advantages over traditional premixed insulin regimens [21–23]. In summary, our experiments provide a molecular-level model for understanding how this particular combination of insulin analogs succeeds clinically.

## ABBREVIATIONS

AU: Absorbance unit
DLS: Dynamic light scattering
NPH: Neutral protamine Hagedorn
PD: Pharmacodynamic
PDI: Polydispersity index
PK: Pharmacokinetic
R_h_: Hydrodynamic radius
SEC: Size-exclusion chromatography
tris: Tris (hydroxymethyl) aminomethane
Zn: Zinc
